# Effect of Central Line Duration and Other Risk Factors on Central Line-Associated Bloodstream Infection in Severe Adult Burns Patients at a Large Tertiary Referral Burns Centre: A 5-Year Retrospective Study

**DOI:** 10.3390/ebj3010003

**Published:** 2022-01-11

**Authors:** Alexandra Miller, Elizabeth Vujcich, Jason Brown

**Affiliations:** Royal Brisbane & Women’s Hospital, Brisbane, QLD 4029, Australia; elizabeth.vujcich@health.qld.gov.au (E.V.); jason.brown@health.qld.gov.au (J.B.)

**Keywords:** central line, sepsis, burns

## Abstract

Central line-associated bloodstream infection (CLABSI) and catheter-related bloodstream infection (CLABSI with a positive catheter tip culture, CRBSI) are preventable causes of morbidity and mortality for severe adult burns patients. Routine central line changes as a CLABSI prevention strategy in burns patients is controversial due to the paucity of evidence to guide the appropriate timing of line changes. This study aimed to address this evidence gap by investigating risk factors associated with central line sepsis, including the duration of central line insertion, in a population of severe adult burns patients (burns involving ≥20% total body surface area (TBSA)) admitted to the Royal Brisbane and Women’s Hospital Intensive Care Unit over five years (2015–2019 inclusive). On multivariate analysis, central line duration and burn TBSA were identified as independent risk factors for CLABSI, with central line duration the most significant predictor (*p* = 0.0008; OR 1.177, 95% CI 1.072–1.299). No risk factor independently predicted CRBSI. CLABSI detection occurred a median of 8.5 days (IQR 6.0–11.0) post central line insertion. These findings suggest further research to assess the efficacy of routine line changes prior to the at-risk period of 6–11 days post central line insertion in reducing CLABSI in severe adult burns patients may be beneficial.

## 1. Introduction

Central line-associated bloodstream infection (CLABSI) and catheter-related bloodstream infection (CLABSI with a positive catheter tip culture, CRBSI) are important causes of morbidity and mortality for severe adult burns patients [1,2,3,4,5,6,7] and contribute significantly to the health care costs of adult burns centres [1,2,3]. Despite burns patients’ inherent high risk of infection, CLABSI and CRBSI within the burns patient population remain preventable.

It is well recognised that burns patients are vulnerable to developing infection. Compared with other intensive care unit (ICU) patients, burns patients are 2–3 times more likely to develop CLABSI [2,4,8,9,10,11]. Having lost their protective skin barrier and with an altered humoral and cellular immunity, severe burns patients are immunocompromised [5,6,12,13]. These patients also require prolonged hospital and ICU admissions [1,14,15], extended durations of central access [1,11,14] with line sites often in close proximity to colonised wounds [4,11,14,15,16], and undergo multiple surgical procedures [1,14,15] and dressing changes [14], all of which predispose to infection. Within this already vulnerable population, factors proposed to further increase CLABSI risk include extremes of age [17], comorbidities [18,19], greater total body surface area (TBSA) of the burn [6,20,21], deeper burns [6], central line insertion through burned skin [11,22], and mechanisms of injury involving gasoline, fire, and gunpowder [6].

However, despite this inherent high risk of infection, CLABSI and CRBSI within the burns patient population remain preventable. Various strategies have been suggested to decrease CLABSI rates in burns patients with varying levels of success. These include careful cannulation site selection (such as avoiding femoral cannulation sites [4,9,23,24] and areas in close proximity to burn wounds [11]), alcohol impregnated caps [15,25,26,27,28,29], chlorhexidine dressings [1,30], antibiotic-coated catheters [25,26,27,28,29], and staff education regarding central line site cares [1,31,32].

The implementation of routine central line changes as a CLABSI prevention strategy remains controversial in burns patients [1]. In the general ICU population, the evidence strongly recommends against routine line changes [33,34,35,36], which is reflected in guidelines published by the National Health and Medical Research Council, Centers for Disease Control and Prevention, and other health agencies [33,34,37,38]. However, in burns patients, studies are conflicting, and practices vary widely in the absence of burns-specific guidelines [5,15,39,40,41]. These differing findings may be due, in part, to the paucity of evidence to guide the appropriate timing of routine line changes.

This study aimed to add to the body of evidence by investigating the risk factors associated with central line sepsis in a population of severe adult burns patients (burn TBSA ≥20%) at a large tertiary referral burns centre and, in particular, to determine whether central line sepsis is more likely to occur after a certain duration of central line insertion to better guide the timing of routine central line changes.

## 2. Materials and Methods

### 2.1. Data Collection

Retrospective data were collected from 121 adult patients with ≥20% TBSA burns who were admitted to the ICU at the Royal Brisbane and Women’s Hospital (Brisbane, Australia) from 2015 to 2019 inclusive. Data collected included age, gender, comorbidities, cause of burn, percentage burn TBSA, central line site, and duration. Each central line was also analysed to determine whether it met criteria for CLABSI or CRBSI. All central lines were placed under sterile conditions using the Seldinger technique and covered with a sterile dressing. In accordance with hospital guidelines, central lines were accessed using a strict aseptic non-touch technique and dressings were changed at least weekly unless otherwise clinically indicated.

### 2.2. Definitions of CLABSI and CRBSI

CLABSI was defined in accordance with the definition recognised by the Australian Commission on Safety and Quality in Health Care [42] as follows.

A laboratory-confirmed blood stream infection where:

1. A central line has been in place for >48 h on the date of the event;
2. A recognised bacterial or fungal pathogen is cultured from one or more blood cultures; and
3. The organism cultured from blood is not related to an infection at another site.

Cultures of potential contaminant organisms, including *Corynebacterium* spp., coagulase negative staphylococci, and *Propionibacterium* spp., were excluded from the analysis in accordance with Australian Commission on Safety and Quality in Health Care guidelines [42].

CRBSI was defined as a CLABSI with a positive catheter tip semi-quantitative culture (>15 colony-forming units) of the same microorganism and antimicrobial susceptibility as the blood culture.

These definitions are consistent with those used in previous studies of CLABSI in burns patients [2,4,5,20,40,43]. Systemic features of infection such as fever were not included in the above definitions due to the invariable presence of these features in severe burns patients secondary to the systemic inflammatory response in the absence of infection [2,44,45,46,47].

Where CLABSI was identified in a patient with more than one central line in situ, the responsible catheter was determined to be the catheter with a positive tip culture matching the blood culture result or, where this was not available, the central line left in situ for the longest duration.

### 2.3. Statistical Analysis

Statistical analysis was performed using GraphPad Prism (version 8.4.2 for macOS) and SPSS (version 28.0.0.0 for macOS). Student’s *t*-test, Mann–Whitney U test, χ^2^ analysis, and Fisher’s exact test were used where appropriate in the univariate analysis of risk factors for CLABSI and CRBSI. The normality of variables was assessed by graphing data on a histogram and using the Anderson–Darling test. A multiple logistic regression was then performed to identify factors independently associated with CLABSI and CRBSI. Variables entered into the multivariate analysis were those with a *p*-value of ≤0.1 on univariate analysis. In the multivariate analysis, *p* < 0.05 was considered statistically significant.

## 3. Results

### 3.1. Demographic Analysis

A total of 343 central lines (2442 line days) were analysed from 121 patients. The mean age of patients studied was 40.01 ± 16.42 years, with a larger proportion of male (n = 89, 73.55%) than female (n = 32, 26.45%) patients. Flame burns were the most common (n = 108, 89.26%). Further, 10 patients (8.26%) had comorbidities associated with potential for immunocompromise, including diabetes, malignancy, or history of steroid use. The median burn TBSA was 40% (IQR 28.50–58.50).

The median number of central lines placed per patient was two lines (IQR 1–4), with a median duration of seven days (IQR 5–9). The most common site for central line placement was femoral (n = 188, 54.81%), followed by internal jugular (n = 100, 29.15%) and subclavian (n = 55, 16.03%).

Criteria for CLABSI were met by 23.14% of patients (n = 28) and 12.83% of central lines (n = 44) during admission. Criteria for CRBSI were met by 6.61% of patients (n = 8) and 2.62% of central lines (n = 9). Total incidence of CLABSI was 18.02 infections per 1000 catheter days and total incidence of CRBSI was 3.69 infections per 1000 catheter days. *Klebsiella pneumoniae* and *Pseudomonas aeruginosa* were the most common pathogens identified on catheter tip culture in CRBSI (see Table 1).

### 3.2. Univariate Analysis of Risk Factors

Univariate analysis found that burn TBSA (*p* = 0.0078) and central line duration (*p* = 0.0006) were significant predictors for patients who developed CLABSI (see Table 2).

**Figure 1:**
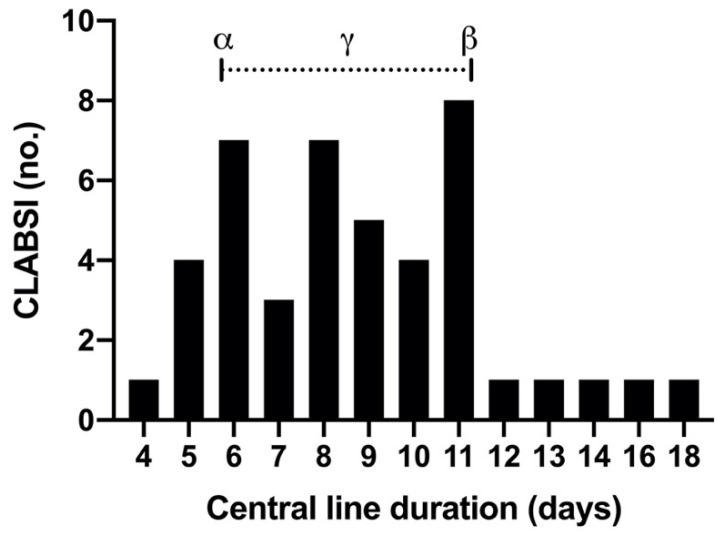
CLABSI frequency by central line duration. CLABSI frequency (no.) increases with central line duration (days); CLABSI occurred at a median of 8.5 days (γ) (IQR 6.0 [α]−11.0 [β]) post central line insertion.

The interval between central line insertion and CLABSI detection ranged from four to 18 days, with a median of 8.5 days post line insertion (IQR 6.0–11.0) (see Figure 1). Cause of burn, cannulation site, age, gender, and comorbidities associated with immunocompromise did not predict CLABSI.

In the CRBSI patient group, central line duration (*p* = 0.0475) and age (*p* = 0.1038) reached the significance threshold for inclusion in the multivariate analysis (see Table 3). Median central line duration prior to CRBSI detection was 10 days (IQR 6.50–10.50).

### 3.3. Multivariate Analysis of Risk Factors

In the CLABSI patient group, the multivariate analysis reflected the univariate analysis, with central line duration and burn TBSA identified as independent risk factors for CLABSI (see Table 4). Central line duration was the most significant predictor of CLABSI (*p* = 0.0008; OR 1.177, 95% CI 1.072–1.299).

For the CRBSI patient group, no risk factor independently predicted CRBSI (see Table 5).

## 4. Discussion

Central lines play a vital role in the resuscitation and acute care of severe burns patients. However, they also provide a route through which microorganisms can gain access to a compromised immune system, resulting in significant morbidity and mortality. This study aimed to determine which factors independently predict central line sepsis in severe burns patients, with a view that these factors could be targeted to reduce the incidence of sepsis in this vulnerable patient group.

CLABSI and CRBSI were assessed separately for predictors of sepsis. The rationale for this separate enquiry was explored in the study by Sihler et al. (2010) which argued that CRBSI is a more accurate reflection of central line infection. While CLABSI assumes the central line is the default source of infection if no other site is found, CRBSI requires proof of catheter involvement in the infective process [43]. While most studies evaluating predictors of central line sepsis have focused upon CLABSI to date, these findings may attribute infections caused by other sources to central line sepsis, resulting in an inaccurate risk factor analysis.

The incidence of 23.14% of patients with CLABSI in this study is comparable to other studies of severe burns patients in which a range of 17–49% has been reported [1,3,7,9,48,49,50,51,52]. This is much higher than results reported for ICU patients in general [6] and can be attributed to burns patients’ comparatively greater immunocompromise [13], prolonged admissions [1,14,15], extended durations of central access [1,11,14], and contaminated wound sites [4,11,14,15,16].

The much lower incidence of CRBSI (6.61% of patients) compared with CLABSI reported in this study is also reflected in other studies [43]. The suggestion from Sihler et al. (2010) is that this lower incidence is more truly reflective of the rates of central line sepsis as it excludes infections attributable to other sources captured in CLABSI rate [43]. However, in this study, the low incidence of CRBSI may also reflect the infrequency with which central line tips were sent for culture (26.24% of all central lines), potentially resulting in CRBSIs passing undetected.

On both univariate and multivariate analyses, greater burn TBSA and central line duration were predictors of CLABSI, with central line duration found to be the strongest predictor. These findings are supported by other studies in the burns patient population [4,6,20,21,53]. Greater burn TBSA contributes to higher rates of central line sepsis by increasing the likelihood of central line insertion through contaminated skin, patient immunocompromise, longer ICU admissions, and extended durations of central line insertion, which provide an opportunity for colonisation of the line and its surrounding skin. While the majority of central lines were placed femoral to avoid line insertion through burned skin, a site reported in previous studies to increase the risk of septic complications [4,9,23,24], line site was not found to be a predictor of CLABSI in this study.

One could infer from these findings that if central line duration was reduced by introducing routine line changes, the incidence of CLABSI could also be reduced. In the general ICU population, the evidence strongly recommends against routine line changes [33,34,35,36], which is reflected in guidelines published by the National Health and Medical Research Council, Centers for Disease Control and Prevention, and other health agencies [33,34,37,38]. However, burns patients are not reflective of the broader ICU population, particularly regarding their increased risk of central lines sepsis [36,53] and longer duration of line placement [1]. Accordingly, these patients require a burns-specific approach.

Unlike those studies of general ICU patients, studies exploring the routine line changes in the burns population have produced mixed results [1]. In the work of King et al. (2007), reducing the frequency of routine line changes from every three days to every four days increased the incidence of central line sepsis [5]. Sood et al. (2017) demonstrated a reduction of the CLABSI rate to zero by implementing routine lines changes every seven days in combination with other CLABSI prevention strategies [1]. However, other studies have found an increased frequency of CLABSI associated with routine line changes [16], or no difference in CLABSI rates with increasing central line duration [4,9].

The reason for these conflicting findings may be due, in part, to the paucity of randomised control trial evidence to guide the appropriate timing of routine line changes. For example, in the work of King et al. (2007), the decision to change lines at days three and four was directed by institution practice [5]. In the current study, CLABSI occurred at a median of 8.5 days post central line insertion (IQR 6.0–11.0). This is similar to findings of previous studies. Sood et al. (2017) [1] and Ramirez-Blanco et al. (2017) [6] found that CLABSI occurred a median of six days post insertion. Sheridan et al. (2006) demonstrated that catheter-related sepsis increased markedly after 10 days in burns patients [54]. Collectively, these results could guide future research into the efficacy of routine line changes prior to this at-risk period (6–11 days post central line insertion) in reducing the incidence of central line sepsis in severe burns patients.

In contrast to the above findings for CLABSI, there were no independent risk factors found to predict CRBSI. This unexpected finding may be explained by the small sample size (n = 8) secondary to the low rate of central line tips sent for culture, which limited the instances of sepsis which could be defined as CRBSI. Alternatively, it may be a true finding, in which case routine line changes may not lead to any appreciable reduction in CRBSI incidence. Further studies with higher rates of central line tips sent for culture are required to determine the significance of this finding.

This study has a number of limitations. The data analysed in this study were drawn from one tertiary referral centre and thus findings may not be reflective of the wider burns patient population. As discussed above, the sample size for the CRBSI analysis was limited by the small percentage of catheter tips sent for culture. Future studies would need to address this significant limitation by ensuring catheter tips are routinely sent for culture. This study also did not implement or directly evaluate the efficacy of interventions to reduce central line sepsis rates, such as routine line changes or specific central line site nursing care.

Despite these limitations, however, this study provides an important preliminary analysis of risk factors for central line sepsis in a population of severe adult burns patients. This information will be useful in designing future research to target questions regarding the efficacy of routine line changes in reducing CLABSI rates.

## Figures and Tables

**Table 1:** CLABSI and CRBSI–organisms and multidrug resistance.

Organisms	CLABSI (% MDR ^1^)	CRBSI (% MDR ^1^)
*Pseudomonas aeruginosa*	13 (23.1%)	3 (66.7%)
*Acinetobacter baumanii*	11 (45.5%)	1 (0%)
*Klebsiella pneumoniae*	7 (14.3%)	3 (0%)
*Enterobacter cloacae*	3 (33.3%)	1 (100%)
*Serratia marcescens*	3 (0%)	0
*Enterobacter aerogenes*	2 (0%)	0
*Proteus mirabilis*	2 (0%)	0
*Candida albicans*	1 (0%)	1 (0%)
Other ^2^	14 (0%)	0

^1^ Multidrug resistant–resistant to at least 3 different antimicrobial classes. ^2^ *Lactobacillus* sp., *granulicatella (abiotrophia) adiacens*, *klebsiella oxytoca*, *pseudomonas chlororaphis*, *bacteroides thetaiotaomicron*, *bacteroides stercoris*, *candida glabrata* complex, *candida dubliniensis*, *staphylococcus aureus*, *veillonella parvula*, *bacillus cereus*, *enterococcus faecium*, *enterococcus faecalis*, *escherichia coli*.

**Table 2:** CLABSI–univariate analysis of risk factors.

Variables	CLABSI (n = 44 Central Lines; 28 Patients)	Non-CLABSI (n = 299 Central Lines; 93 Patients)	*p* Value	Odds Ratio (95% CI)
Age (years) (mean+/−SD)	38.71+/−16.73	40.40+/−16.40	0.6364	
Female (no)	10 (35.7%)	22 (23.7%)	0.2047	1.7930 (0.6916–4.5790)
Comorbidities assoc. with immuno- compromise	4 (14.3%)	6 (6.5%)	0.2376	2.417 (0.7168–8.4940)
Cause of burn				
Flame	24 (85.7%)	84 (90.3%)	0.1360	
Electrical	1 (3.6%)	6 (6.5%)		
Scald	2 (7.1%)	1 (1.1%)		
Combination	0	2 (2.2%)		
Unclear	1 (7.1%)	0		
Central line site (no)				
Femoral	26 (59.1%)	162 (54.2%)	0.7913	
Internal jugular	11 (25%)	89 (29.8%)		
Subclavian	7 (15.9%)	48 (16.1%)		
Central line duration (days) (median, IQR)	8.50 (6.00–11.00)	7.00 (5.00–9.00)	0.0006 ^1^	
TBSA (%) (median, IQR)	47.00 (39.25–70.38)	36.00 (25.25–56.75)	0.0078 ^2^	

^1^*p* < 0.001. ^2^ *p* < 0.01.

**Table 3:** CRBSI–univariate analysis of risk factors.

Variables	CRBSI (n = 9 Central Lines; 8 Patients)	Non-CRBSI (n = 334 Central Lines; 113 Patients)	*p* Value	Odds Ratio (95% CI)
Age (years) (mean+/−SD)	53.00 (35.50–60.50)	37.00 (24.50–51.00)	0.1038	
Female (no)	4 (50%)	28 (24.8%)	0.1180	3.0360 (0.8301–10.8700)
Comorbidities assoc. with immuno- compromise	0	10 (8.8%)	0.2376	2.417 (0.7168–8.4940)
Cause of burn				
Flame	8 (100%)	100 (88.5%)	1.0000	
Electrical	0	7 (6.2%)		
Scald	0	3 (2.7%)		
Combination	0	2 (1.8%)		
Unclear	0	1 (0.9%)		
Central line site (no)				
Femoral	5 (55.6%)	183 (54.8%)	1.0000	
Internal jugular	3 (33.3%)	97 (29%)		
Subclavian	1 (11.1%)	54 (16.2%)		
Central line duration (days) (median, IQR)	10.00 (6.50–10.50)	7.00 (5.00–9.00)	0.0475 ^1^	
TBSA (%) (median, IQR)	50.00 (28.88–75.18)	40.00 (28.50–57.50)	0.3892	

^1^*p* < 0.05.

**Table 4:** CLABSI–multivariate analysis of risk factors.

Variables	*p* Value	Odds Ratio (95% CI)
Central line duration	0.0008 ^1^	1.1770 (1.0720–1.2990)
TBSA	0.0490 ^2^	1.0160 (1.0000–1.0320)

^1^*p* < 0.001; ^2^ *p* < 0.05.

**Table 5:** CRBSI–multivariate analysis of risk factors.

Variables	*p* Value	Odds Ratio (95% CI)
Age	0.4184	1.0170 (0.9764–1.0590)
Central line duration	0.0919	1.1750 (0.9716–1.4180)

## Data Availability

The data presented in this study are available on request from the corresponding author. The data are not publicly available to protect patient confidentiality.
